# Influence of the Polysaccharide Capsule on the Bactericidal Activity of Indolicidin on S*treptococcus pneumoniae*

**DOI:** 10.3389/fmicb.2022.898815

**Published:** 2022-05-13

**Authors:** Natalha T. Waz, Sheila Oliveira, Raquel Girardello, Nilton Lincopan, Giovana Barazzone, Thais Parisotto, Anders P. Hakansson, Thiago Rojas Converso, Michelle Darrieux

**Affiliations:** ^1^Laboratório de Biologia Molecular de Microrganismos, Universidade São Francisco, Bragança Paulista, Brazil; ^2^Laboratório de Resistoma e Alternativas Terapêuticas, Instituto de Ciências Biomédicas, Universidade de São Paulo, São Paulo, Brazil; ^3^Laboratório de Desenvolvimento de Vacinas, Instituto Butantan, São Paulo, Brazil; ^4^Division of Experimental Infection Medicine, Department of Translational Medicine, Lund University, Malmo, Sweden

**Keywords:** capsular polysaccharide, indolicidin, *Streptococcus pneumoniae*, antimicrobial peptides, AMP resistance

## Abstract

*Streptococcus pneumoniae* is a pathogen responsible for high morbidity and mortality worldwide. The polysaccharide capsule confers protection against phagocytosis and influences many aspects of pneumococcal pathogenesis. The capsular polysaccharides (CPS) are highly immunogenic and exhibit great structural variability, with more than 100 serotypes described so far. Antimicrobial peptides (AMPs) are an important part of the innate defense mechanisms against many pathogens. Indolicidin is a cationic AMP produced by bovine neutrophils, with bactericidal effects against several bacteria. CPS has been shown to interfere with the ability of AMPs to kill pneumococci, but the effects of capsule variability on susceptibility to indolicidin have not been explored. The present work determined the effects of capsule on resistance to indolicidin *in vitro*. Using a bactericidal plate assay, we observed that different pneumococcal serotypes exhibited variable resistance to indolicidin, which correlated with the capsule net charge. Interestingly, the effect of capsule expression on resistance to indolicidin was dependent on the serotype; bacteria with lower zeta potential were more resistant to indolicidin when capsule was present, while those with less negative surface charge were more resistant in the absence of capsule. The addition of purified CPS partially rescued the bacteria from the bactericidal effects of indolicidin, while the addition of anticapsular antibodies accentuated the peptide’s bactericidal action, suggesting a possible new protective mechanism induced by polysaccharide-based pneumococcal vaccines.

## Introduction

*Streptococcus pneumoniae* (pneumococcus) infections are a worldwide public health problem. Every year, over one million people, mostly children and the elderly in developing countries, die of diseases caused by this organism (Feldman and Anderson, 2020). The pneumococcus is a Gram-positive microorganism that colonizes the human upper respiratory tract asymptomatically. When changes of the nasopharyngeal environment occur, such as during concomitant infection with respiratory viruses, most commonly influenza virus or respiratory syncytial virus, or due to deficiency in host defense mechanisms (McCullers, 2014), *S. pneumoniae* can invade disseminate to other sites such as the lungs, meninges, and blood, and cause an intense inflammatory response that can be fatal (Kadioglu et al., 2008).

The pneumococcal cell envelope is composed of three main structures: the innermost plasma membrane, formed by a lipid bilayer; the cell wall consisting of peptidoglycans and teichoic and lipoteichoic acid, which anchors several surface proteins; and the polysaccharide capsule in the outermost portion, which is quite variable in thickness and chemical composition (Kadioglu et al., 2008).

The polysaccharide capsule is an important virulence factor, involved in the evasion of complement protein deposition and consequent phagocytosis during human host invasion (Hyams et al., 2010). Due to its structural variability, the composition of the capsule is used as a classification criterion for the bacterium and this classification currently contains more than 100 individual serotypes (Ganaie et al., 2020).

Capsular polysaccharides (CPS) are highly immunogenic and form the basis of the pneumococcal vaccines currently in use. Pneumococcal conjugated vaccines are effective in the control of invasive diseases and are also able to reduce nasopharyngeal colonization, the first stage of infection, that is a prerequisite for all diseases caused by the pneumococcus (Briles et al., 2019).

Antimicrobial peptides (AMP) are low molecular mass proteins capable of inhibiting the growth of bacteria, viruses, and fungi. They are part of the innate immune system of several classes of living organisms (Zasloff, 2002). Most AMPs are cationic and amphipathic and act by destabilizing the membranes of microorganisms (Kuppusamy et al., 2019). To date, 116 different AMP have been identified in humans and can be found in different tissues and expressed on the skin, eyes, and mucosal surfaces such as oral cavity, intestines, and urinary tract (Wang et al., 2016).

The positive charge of antimicrobial peptides is crucial for their action against bacteria. Unlike eukaryotic plasma cell membranes, which are composed of neutral lipids, the cytoplasmic membranes of Gram-positive and Gram-negative bacteria are rich in highly electronegative lipids, such as phosphatidylserine (PS), cardiolipin (CL), or phosphatidylglycerol (PG). These structures give the bacterial membrane a negative charge, which attracts positively charged peptides. The interaction of AMPs with the bacterial membrane in most cases leads to the formation of pores and subsequent rupture of the microbial cell, but this interaction may also lead to inhibitory effects on bacterial metabolism and translation (Brogden, 2005; Lazzaro et al., 2020).

In addition to their positive charge, antimicrobial peptides have a high content of hydrophobic residues, such as tryptophan, which allows the AMP to penetrate the interfacial regions of lipid bilayers, facilitating the interaction of antimicrobial peptides with the underlying bacterial cell membrane (Lai and Gallo, 2009).

Besides a direct bactericidal effect, AMPs can exert activities in other ways, such as being pro-apoptotic, anticarcinogenic, pathogenic toxin neutralizers, or acting as immunomodulators (Siqueiros-Cendon et al., 2014; Mangoni et al., 2016).

To counteract AMPs’ bactericidal activities, bacteria have evolved an arsenal of resistance mechanisms, including efflux pumps and transport systems, AMP sequestration and inactivation, competition, and envelope modifications that promote AMP repulsion or inhibit their ability to bind to the cell membrane (Assoni et al., 2020). These latter modifications usually affect surface charge, thereby limiting the interaction between the bacterial membranes and the positively charged AMPs. One defense mechanism used by the pneumococcus is its polysaccharide capsule, which shows great variability in chemical composition between serotypes, resulting in structures that range from highly negative to those closer to neutrality. Serotypes with the most negative capsular structures have been associated with increased resistance to *in vitro* phagocytosis by neutrophils and an increased ability to colonize the host (Li et al., 2013).

Indolicidin is an antimicrobial peptide belonging to the cathelicidin family, secreted by neutrophils during their activation (Braff et al., 2005). It displays inhibitory activity against bacteria, fungi, viruses, and cancer cells, as well as a possible chemotactic activity for immune cells such as neutrophils, monocytes, and T lymphocytes (Vegh et al., 2011).

Indolicidin is a short linear peptide, composed of 13 amino acid residues—(ILPWKWPWWPWRR-Am). The C-terminal region of the molecule is responsible for the antimicrobial action and this region undergoes a process called amidation, with loss of the −OH group of the carboxyl-terminal portion (−COOH), which is replaced by an amine group (Rozek et al., 2000; Friedrich et al., 2001).

The action of indolicidin occurs through the rupture of bacterial membranes (Falla et al., 1996; Rozek et al., 2000). In Gram-negative bacteria, indolicidin has been shown to rapidly permeate the cell wall to reach its target, the plasma membrane, where it forms channels that lead to bacterial rupture (Galdiero et al., 2016). A similar effect was observed in *Streptococcus pneumoniae* and *Staphylococcus aureus* (Gram-positive bacteria), suggesting that indolicidin is able to cross the thick barrier of the cell wall and promote plasma membrane destabilization (Jindal et al., 2015). The positive charge of the AMP combined with its high hydrophobic content and high concentration of tryptophan (Jindal et al., 2015, 2017) contribute to the bactericidal activity. It has also been demonstrated that indolicidin enters bacterial cells, binds to the DNA double helix, thereby preventing replication and transcription and amplifying its antimicrobial action (Ghosh et al., 2014; Jindal et al., 2015).

Previous data from our group show that pneumococci are partially resistant to the lytic action of indolicidin, thanks to the presence of pneumococcal surface protein A (PspA; Milani et al., manuscript in preparation), a surface-exposed protein able to bind to lactoferrin and prevent its lytic bactericidal effects (Hakansson et al., 2001; Shaper et al., 2004; Andre et al., 2015). However, the contribution of the polysaccharide capsule, a structure known to affect bacterial resistance to phagocytosis and AMPs such as defensins, to indolicidin has not yet been investigated. In this study, we evaluated the influence of the polysaccharide capsule in protecting the bacteria against the action of indolicidin and whether the protective effect of the capsule varies based on its composition. Using wild type and mutant pneumococcal strains, as well as adding purified polysaccharides and anticapsular antibodies, we investigated the role of capsule in resistance to killing by indolicidin.

## Materials and Methods

### Bacterial Strains and Culture

The bacterial strains used in the study are presented in Table 1. Bacterial strains were grown in Todd-Hewitt medium containing 0.5% yeast extract (THY; purchased from Kasvi) and stocks were kept frozen at −80°C.

**Table 1:** *Streptococcus pneumoniae* isolates used.

Strain	Serotype	Source	References
St 245/00	14	IAL^1^	Goulart et al., 2011
A66.1	3	UAB^2^	Avery et al., 1944; Briles et al., 2000
D39	2	UAB^2^	Avery et al., 1944
TIGR 4	4	UAB^2^	Tettelin et al., 2001
AM 1000^&^	_	LU^3^	Magee and Yother, 2001
HR 1001^£^	_	LU^3^	Thamadilok et al., 2016
St 0603	6B	BCH^4^	Malley et al., 2006
P1079	1	UFG^5^	Pimenta et al., 2006; Goulart et al., 2011
P1031	23F	UFG^5^	Pimenta et al., 2006; Goulart et al., 2011
P1153	9V	UFG^5^	Pimenta et al., 2006
P69	10A	IAL^1^	Pimenta et al., 2006; Goulart et al., 2011

1 IAL – Instituto Adolfo Lutz.

2 UAB – University of Alabama at Birmingham, United States.

3 LU – Lund University, Malmo, Sweden.

4 BCH – Boston Children’s Hospital, United States.

5 UFG – Universidade Federal de Goiás, Brazil.

& AM1000 is derived from D39.

£ HR1001 is derived from TIGR4.

The day before each experiment, 20 μl of the frozen stock of each pneumococcal strain were plated on blood agar and incubated overnight at 37°C under microaerophilic conditions.

The next morning, bacterial colonies were transferred to 7 ml of THY media and incubated at 37°C and the optical density (O.D._600nm_) was monitored until reaching an O.D._600nm_ between 0.3 and 0.4.

### Bactericidal Assay

Bacterial cultures (5 ml) at the desired O.D._600nm_ were transferred to another tube and the bacteria were pelleted by centrifugation and washed with 5 ml of sterile PBS solution and resuspended in 2 ml of sterile PBS. Next, the bacterial suspensions were incubated in the presence of increasing concentrations of indolicidin (ANASPEC, code AS-60999), ranging from 7.5 to 120 μg/ml, diluted in phosphate buffered saline (PBS) to a final volume of 100 μl. The untreated control samples were incubated with PBS alone.

The samples were incubated at 37°C for 1 h, serially diluted, and plated on blood agar. The number of bacteria surviving treatment was calculated as the colony forming units per ml (CFU/ml) for each group compared with the untreated control.

### Effect of the Polysaccharide Capsule on the Bactericidal Action of Indolicidin

To assess the contribution of the polysaccharide capsule to the lytic action of indolicidin on pneumococci, wild type and isogenic non-capsular mutant strains were subjected to treatment with indolicidin as described above. The effect of purified polysaccharides on indolicidin activity was investigated by mixing 10 μg of purified CPS of serotype 6B (ATCC) with indolicidin 15 min prior to incubation with the bacteria.

### Mouse Immunization

The animal experiments were approved by the São Francisco University Animal Ethics Committee (protocol 003.04.2021). Female BALB/c mice (Obtained from CEMIB – UNICAMP, Brazil) were immunized i.p. with 3 doses of 10 μg of CPS 1 and 6B (ATCC) using 100 μg of Al(OH)_3_ as an adjuvant, in a final volume of 0.5 ml, at 12 days intervals. Blood was collected through retroorbital bleeding and serum from coagulated blood was stored at −20°C.

### Effect of Anticapsular Antibodies on the Bactericidal Action of Indolicidin

To determine the ability of anticapsular antibodies to block the potential protective action of the capsule against the effects of indolicidin, wild-type pneumococci P1079 (serotype 1) and St 0603 (6B) were incubated in the presence of serum from mice immunized with purified CPS 1 and 6B, respectively, 15 min prior to treatment with indolicidin. The control group was incubated in presence of serum from mice injected with adjuvant alone in saline. The number of bacteria surviving treatment was determined in each group and compared with the control.

### Zeta Potential Measurement

The strains were grown on 5% sheep blood agar for 24 h under microaerophilic conditions as described above. A bacterial suspension at 1.5 × 10^8^ CFU/ml was prepared immediately after cultivation and washed twice by centrifugation at 3,000 rpm for 5 min in a 1 mM NaCl solution. The precipitate was diluted in 2 ml of 1 mM NaCl and the experiment was performed in Zeta Plus Potential Analyzer (Brookhaven Instruments Corporation, Holtsville, NY), starting with bacterial particle size measurement using Particle Sizing software (Brookhaven Instruments Corporation, Holtsville, NY), followed by measurement of zeta potential using Zeta Plus software (Brookhaven Instruments Corporation, Holtsville, NY). To evaluate the effect of antibody treatment on surface charge, the zeta potential of type 1 and type 6B pneumococci were measured after opsonization with anticapsular antibodies during 1 or 15 min and compared with control serum. The experiments were carried out in duplicates and repeated to confirm the results.

### Statistics

Statistical analyses were performed using the ANOVA followed by Dunnett’s (treatment versus control) or Tukey’s (to compare the differences among different treatments) post-test for multiple comparisons. Pearson’s correlation was used to evaluate the relationship between zeta potential and resistance to indolicidin treatment. Differences were considered statistically significance if *p* ≤ 0.05. The statistical analyses and all graphs were performed using GraphPad Prism 9.

## Results

### Pneumococcal Resistance to Indolicidin Is Influenced by the Capsular Serotype

**Figure 1:**
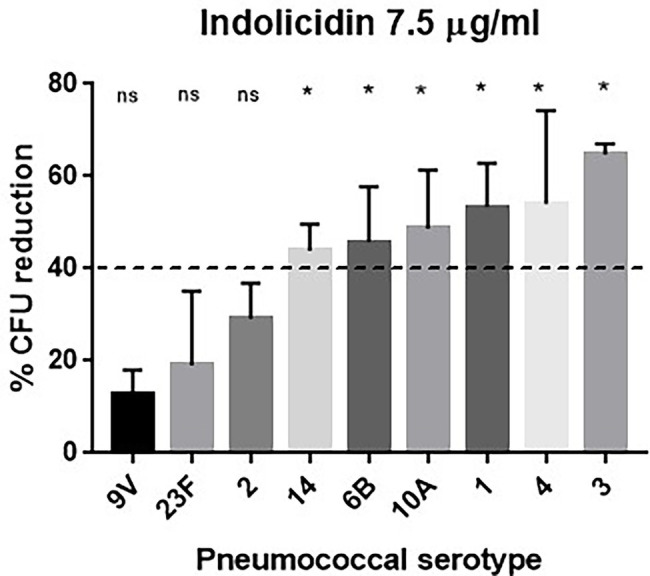
Susceptibility of *Streptococcus pneumoniae* to indolicidin. Bacterial strains of different serotypes were treated with 7.5 μg/ml indolicidin and plated. The percentage of bacterial reduction after treatment is shown for each strain. Statistical analysis was performed using ANOVA with a Dunnet post-test. ^*^p < 0.05 in comparison with untreated control; ns, not significant.

Pneumococci expressing different capsule types (1, 2, 3, 4, 6B, 9 V, 10A, 14, and 23F) were subjected to treatment with increasing concentrations of indolicidin (ranging from 7.5 to 120 μg/ml). As shown in Figure 1, the pneumococcal strains greatly varied in their ability to resist lysis and killing by indolicidin. Serotypes 9 V, 23F, and 2 showed the highest resistance to indolicidin and were only susceptible to concentrations of 15 μg/ml and higher (Supplementary Figure 1). Serotypes 1, 3, 4, 6B, 10A, and 14, on the other hand, were susceptible to indolicidin killing at the lowest concentration, 7.5 μg/ml. Reductions of 40% and higher in bacterial viability after treatment with the lowest dose of indolicidin were considered as sensitive, based on statistical significance in comparison with the untreated control (Supplementary Figure 1).

**Figure 2:**
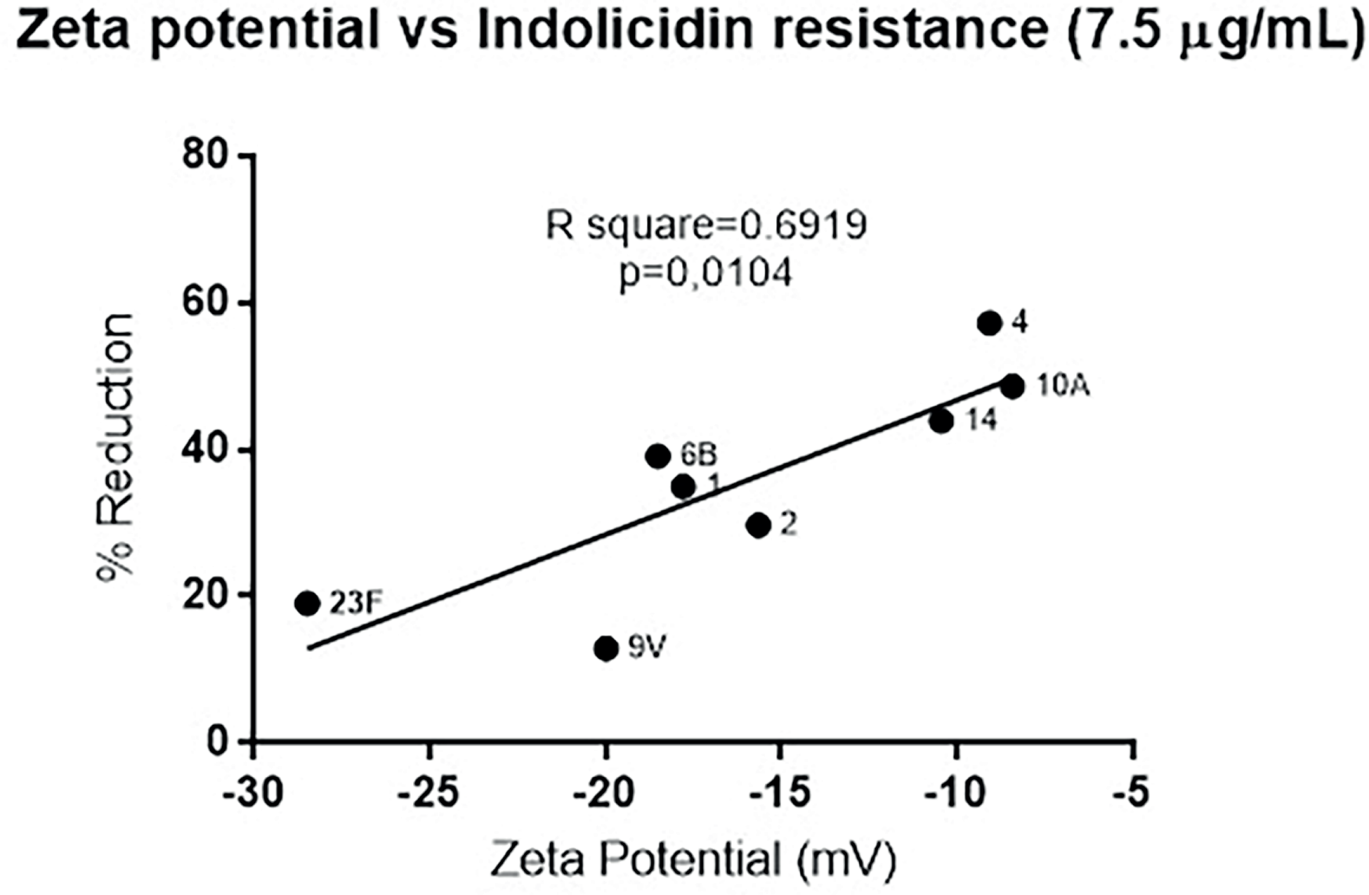
Correlation between of *Streptococcus pneumoniae* surface charge and resistance to killing by indolicidin. Surface charge was determined by calculating the zeta potential of each individual serotype. Resistance is represented by the percent reduction of each bacterial strain after 1 h treatment with 7.5 μg/ml indolicidin. Statistical analysis was performed using Pearson’s comparison.

Since polysaccharide capsules present variations in net charge, the zeta potential of each strain was calculated and plotted against the percent reduction in bacterial viability after indolicidin treatment. The analysis indicates a significant positive correlation between electronegativity and resistance to killing by indolicidin (Figure 2). The exception was serotype 3, which presented around 60% reduction upon indolicidin treatment and a very low zeta potential (not shown).

### The Absence of Capsule Differently Impacts Resistance to Indolicidin

Once the susceptibility of the D39 (serotype 2) and TIGR4 (serotype 4) strains to lysis by indolicidin was established, a comparative analysis was performed using the respective capsule-negative isogenic mutants AM1000 and HR1001.

**Figure 3:**
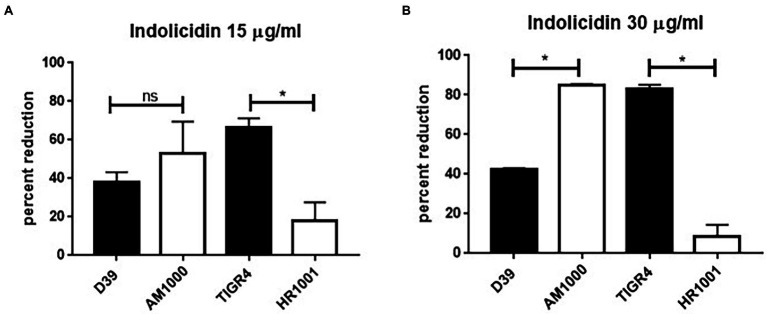
Capsule affects pneumococcal resistance do indolicidin. Wild-type pneumococcal strains D39 (serotype 2) and TIGR4 (serotype 4) and their capsule-negative mutants AM1001 (D39 background) and HR1001 (TIGR background) were treated with indolicidin at 15 μg/ml **(A)** and 30 μg/ml **(B)** and plated. The percent reduction in bacterial survival is shown for each group. Statistical analysis was performed using Student’s *t*-test. ^*^p < 0.05 in comparison with the wild-type strain; ns, not significant.

The absence of capsule had opposite impacts on resistance to indolicidin for D39 and TIGR4 (Figure 3). At the lower concentrations of indolicidin (15 μg/ml), no differences in survival were observed between D39 and its capsule-negative derivative, AM1000 (Figure 3A). However, at a higher concentration of indolicidin (30 μg/ml), the wild-type strain showed an increased resistance against killing, with 40% reduction in comparison with 80% killing in the capsule-negative strain, suggesting a protective effect of the capsule (Figure 3B). On the other hand, the capsule-negative mutant in the TIGR4 background showed an increased resistance to the AMP, indicated by a smaller percentage of bacterial reduction after indolicidin treatment (Figure 3A). This effect was intensified at the higher concentration of indolicidin (30 μg/ml), which caused an 80% reduction in survival of TIGR4, compared with only 14% reduction in the mutant strain (Figure 3B). Taken together, these results suggest that different capsules affect resistance to indolicidin in different ways.

### Effect of Purified CPS on Indolicidin Bactericidal Activity

**Figure 4:**
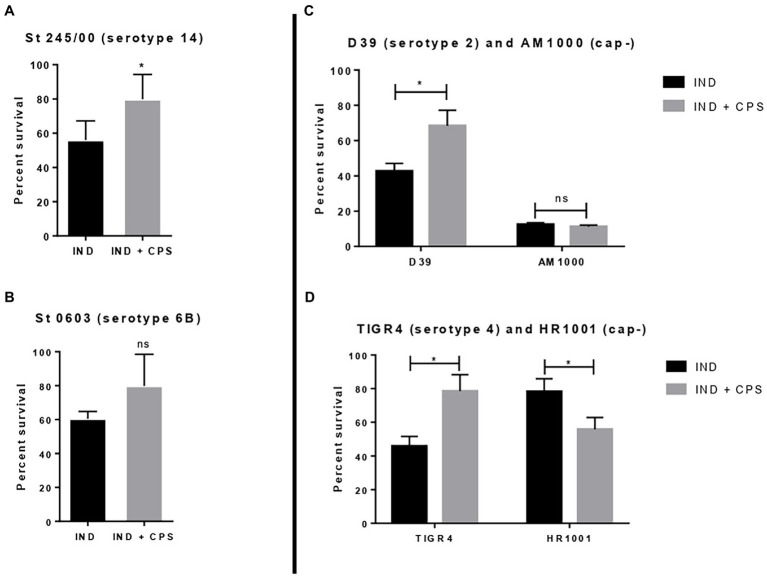
Effect of free capsular polysaccharide on the lytic action of indolicidin. Peumococcal strains St 245/00 **(A)**, St 0603, serotype 6B **(B)**, D39, serotype 2 and its capsule-negative mutant **(C),** and TIGR4 and its capsule-negative mutant **(D)** were treated with indolicidin preincubated with purified CPS 6B. Each bar represents the percent in survival relative to the control group. Statistical analysis was performed using Student’s *t*-test. ^*^p < 0.05 in comparison with indolicidin treatment in absence of CPS. ns, not significant.

Pneumococcal strains St. 245/00 (serotype 14), St. 0603 (serotype 6B), D39 (serotype 2), and TIGR4 (serotype 4, plus their isogenic capsule-negative mutants (AM1000 and HR 1001)) were incubated with indolicidin and purified CPS 6B. The results are shown in Figure 4. The addition of purified CPS 6B protected the strains St 245/00, D39, and TIGR4 from the effect of indolicidin. The strain St 0603 was partially protected from indolicidin action; the group treated with the AMP plus CPS had bacterial counts similar to those of the control group, but not statistically different from the group treated with indolicidin alone. The protective effect of CPS6B was lost when the concentrations of indolicidin were increased (data not shown). Presence of CPS did not affect killing of the capsule-negative strain AM1000 (Figure 4C). Interestingly, for HR 1001, addition of CPS had the opposite effect, rendering the bacteria less resistant to killing by indolicidin (Figure 4D).

### Effect of Anticapsular Antibodies on Indolicidin Action

**Figure 5:**
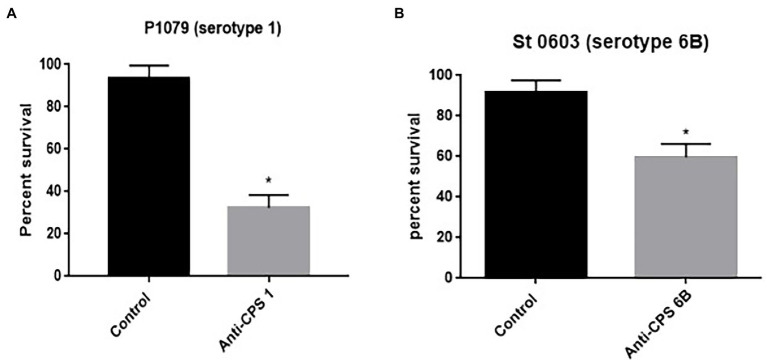
Effect of anticapsular antibodies on the lytic action of indolicidin. Peumococcal strains St 1,079, serotype 1 **(A)** and 0603, serotype 6B **(B)** were incubated with sera from mice immunized with CPS 1 and 6B, respectively, prior to treatment with indolicidin. The control group was incubated with sera from sham immunized mice. The percentage of bacteria surviving treatment is shown for each group. Statistical analysis was performed using Student’s *t*-test. ^*^p < 0.05 in comparison with the control.

To evaluate the effect of anticapsular antibodies over indolicidin killing, pneumococcal strains P1079 (serotype 1) and St. 0603 (serotype 6B), were preincubated with sera from mice immunized with the homologous CPS, followed by treatment with indolicidin. The results of this analysis are shown in Figure 5. Incubation with anti-CPS1 increased bacterial killing by indolicidin in comparison with control sera (Figure 5A). This effect may be attributed to the antibodies concealing protective epitopes in the capsule, thus blocking further interactions with the peptide. A similar effect was observed with antisera against the serotype 6B strain (St 0603; Figure 5B). The zeta potential of the strains after incubation with anticapsular antibodies was also measured at different time points. No changes in surface charge were observed in opsonized bacteria in comparison with the control at any time (not shown).

## Discussion

The polysaccharide capsule plays a central role in pneumococcal pathogenesis, protecting the bacterium against phagocytosis (Paton and Trappetti, 2019). Recent data also demonstrate a role for the capsule in favoring pneumococcal translocation through the vascular endothelium and enhancing intracellular survival—key steps involved in transition from carriage to invasive disease (Brissac et al., 2021). The contribution of capsule to pneumococcal virulence is further emphasized by the high protection observed with the current polysaccharide-based vaccines against invasive diseases (Briles et al., 2019).

However, capsules present high structural and serological diversity; to date, more than 100 pneumococcal serotypes have been identified (Ganaie et al., 2020). In addition, the degree of protection that the polysaccharide capsule confers varies according to the serotype (Geno et al., 2015; Paton and Trappetti, 2019).

In the present study, we investigated the contribution of the capsule to resistance or susceptibility of *Streptococcus pneumoniae* against the lytic action of indolicidin, an antimicrobial peptide belonging to the cathelicidin family.

Initially, *S. pneumoniae* isolates bearing different capsular types (Table 1) were exposed to a 1 h treatment with increasing concentrations of indolicidin. At the end of the treatment, a range of variations in killing was observed. Serotypes 9 V, 23F, and 2 were more resistant to indolicidin when compared with serotypes 1, 3, 4, 10A, and 14. A dose-dependent effect was observed with all strains tested, with a more pronounced bacterial reduction at higher indolicidin concentrations.

To determine if resistance to indolicidin was affected by the surface charge (which is mainly due to the presence of capsule), we measured the zeta potential of the pneumococcal strains and plotted the results against the killing assay data. A strong positive correlation was observed between surface net charge and resistance to the peptide, indicating that surface charge could predict pneumococcal susceptibility to indolicidin *in vitro*. The only exception was the capsule 3 strain, A66.1, which showed high electronegativity and high susceptibility to killing by indolicidin.

A study conducted by Li et al. (2013) investigating the contribution of surface charge to neutrophil mediated killing, also found that more electronegative capsules were related to increased resistance to phagocytosis and an increased ability to colonize the human nasopharynx. The present results further aid in this hypothesis, suggesting that capsule charge can influence several virulence attributes in this bacterium. Furthermore, that same study found contrasting results with a serotype 3 strain, indicating that this capsular serotype behaves differently from the predictions. Interestingly, the capsule type 3 has some unique characteristics, including its non-covalent attachment to the bacterial surface and extensive capsule production, leading to the mucoid phenotype (Luck et al., 2020). These unique features may be related to the often-unexpected results observed with serotype 3 pneumococci in different comparison analysis. The study from Li et al. (2013) has also looked at the differences in capsule switch mutants with a TIGR4 background, and the results were, in general, similar to those of the clinical isolates, with the more electronegative capsules presenting increased carriage prevalence. Other studies using capsule switch mutants have found a correlation between capsule type, interaction with complement system molecules, and invasiveness (Melin et al., 2010; Hyams et al., 2013). Furthermore, Melin et al. (2010) demonstrated that serotype was the most important determinant of pneumococcal resistance to complement deposition and opsonophagocytic killing (Melin et al., 2010). Although the present study did not employ capsule switch variants, it is plausible to infer, based on previous studies, that capsule type is an important predictor of surface charge and resistance to multiple host defense mechanisms.

Next, we sought to determine if the ability to produce capsules affected killing by indolicidin, by comparing two pneumococcal strains of different serotypes with their capsule-negative isogenic mutants. The type 2 strain D39 was significantly more resistant to lysis with a high dose of indolicidin in comparison with its mutant, AM1000. The serotype 4 strain TIGR4, on the other hand, revealed an opposite effect, with the mutant displaying increased resistance to killing in relation to the wild-type counterpart. A possible explanation for this apparent discrepancy may be found on the surface charge of these strains; D39 was highly electronegative, while TIGR4 showed a zeta potential closer to neutrality. Therefore, we postulate that the effect of the capsule over the action of indolicidin will depend on the ability of such polysaccharides to prevent the lytic action of the AMP.

Previous work evaluating resistance of *S. pneumoniae* expressing diverse serotypes to human defensins HNP1-3 reported similar results (Beiter et al., 2008). The unencapsulated TIGR4 was much more resistant to HNP1-3 when compared to the encapsulated strain. For D39, no differences in killing were observed between the wild-type and mutant strains, in the concentrations of AMP tested. Also, a study from Van der Windt et al. (2012) has shown that nonencapsulated pneumococci have increased resistance to neutrophil proteases, which correlates with the enhanced ability of these strains to resist mucosal immunity mechanisms. In the present work, the increased resistance of the wild-type D39 in relation with the mutant was only apparent when higher concentrations of indolicidin were used, suggesting that other factors beyond the capsule may be involved in resistance to the AMP. Since both capsule-negative mutants exhibited similar (and very low) surface charge (−28.59 for AM1000 and − 27.47 for HR 1001, respectively) one possible explanation for the opposite effects of capsule absence on pneumococcal resistance to indolicidin may involve other AMP resistance mechanisms such as surface proteins, cell wall, or membrane modifications. Beiter et al. (2008) has shown that nonencapsulated pneumococcal mutants have increased resistance against killing by neutrophil extracellular trap (NET)-derived components (Beiter et al., 2008), and effect that was aided by the presence anionic of proteins LytA and PgdE. The authors conclude that the capsule could mask these underlying mechanisms that act through preventing AMP activity. Modifications in cell wall, such as those promoted by the dlt operon, have also been shown to induce increased resistance to nisin and gallidermin in pneumococci (Kovacs et al., 2006). These mechanisms could contribute to the individual differences observed between the capsule-negative mutants; however, further experiments are needed to confirm this hypothesis.

Next, we evaluated whether purified CPS could influence killing by the AMP. Addition of purified CPS 6B protected pneumococcal strains D39, TIGR 4, and St 245/00, while the strain St 0603 was partially protected from killing. These results are in accordance with previous work using purified CPS 6B added to sensitive *Klebsiella pneumoniae* cultures, which showed a protective effect against killing by polymyxin B and HNP-1 (Llobet et al., 2008). In that study, protection was observed with negatively charged CPS, but not with cationic or uncharged ones. The authors conclude that the protective effect of CPS against killing by AMPs is dependent on their charge. They also postulate that in presence of AMPs, bacteria may shed negatively charged CPS which can prevent lysis. This capsule shedding could, therefore, serve a dual purpose for pneumococcus, binding to and sequestering AMPs. That same study also showed that purified CPS can directly bind to the AMPs—a possible mechanism involved in protection against the indolicidin that would require further confirmation. In the present study, however, the addition of CPS to the capsule-negative mutant AM100 (derived from D39) did not affect killing by indolicidin, while in the mutant HR1001 (TIGR4 background), the presence of purified CPS increased the AMP bactericidal activity. These results suggest that, in the absence of capsule, other factors like surface proteins and cell wall/membrane modifications may impact the bacterial interactions with the AMP through distinct mechanisms. This suggestion is corroborated by the success of nonencapsulated pneumococcal strains (Nesps) in colonizing the host and causing infections in niches like the middle year and the eye (Keller et al., 2016), which harbor high concentrations of AMPs. This is particularly interesting in the case of HR1001, which showed an increased resistance to indolicidin and to other AMPs described in the literature, like HNP1-3 (Beiter et al., 2008) in comparison with the wild-type TIGR4.

Lastly, we tested the ability of anticapsular antibodies to interfere with indolicidin activity. Anti-CPS1 and anti-CPS6B increased pneumococcal killing by indolicidin, suggesting a possible new protective mechanism induced by polysaccharide-based pneumococcal vaccines. It also indicates that the ability of CPS to reduce killing by indolicidin involves a direct interaction between the polysaccharide and the AMP; the presence of antibodies would limit this interaction, thus abrogating the protective effects of the capsule and allowing the peptide to access the subjacent bacterial membrane, promoting killing. Analysis of zeta potential revealed that anticapsular antibodies did not affect the bacterial surface charge, indicating that other mechanisms are responsible for the observed effects of pre-opsonization on susceptibility to indolicidin.

Habets et al. (2012) compared the susceptibility of several pneumococcal isolates to the human cathelicidin LL-37 and the alpha-defensin HNP-1 and found that carriage isolates were more resistant to the AMPs than clinical isolates. This finding suggests that AMPs act as selection force for pneumococci during colonization—the first step in all pneumococcal diseases.

## Conclusion

Different pneumococcal serotypes exhibited variable resistance to indolicidin, which correlated with the capsule net charge; bacteria with lower zeta potential were more resistant to indolicidin when capsule was present, while those with less negative surface charge were more resistant in the absence of capsule.

Our results are in accordance with previous work using other antimicrobial peptides, indicating that the capsule has broad (however diverse) effects on the lytic activity of CAMPs. Purified CPS was able to protect the pneumococcus against indolicidin killing, while anticapsular antibodies favor the lytic activity of the AMP. We postulate that electronegative CPS protects pneumococci from indolicidin by hijacking the AMP, thus preventing it from reaching the subjacent cell membrane.

## Data Availability Statement

The original contributions presented in the study are included in the article/Supplementary Material, further inquiries can be directed to the corresponding author.

## Author Contributions

MD, TC, and AH designed the study. NW, GB, and SO performed the indolicidin experiments. RG and NL did the zeta potential analysis. NW, SO, and MD drafted the manuscript. NW and TC revised the data. TC and TP performed the statistical analysis. TC and AH revised the text and figures. All authors read and approved the final manuscript.

## Funding

This work was supported by São Paulo Research Foundation (FAPESP, grant number 2020/11037-6), Coordination for the Improvement of Higher Education Personel (Capes, grant number 88887.495999/2020–00), CNPq (grant 400099/2022-5), and Swedish Research Council (VR) grant number 2021-06050.

## Conflict of Interest

The authors declare that the research was conducted in the absence of any commercial or financial relationships that could be construed as a potential conflict of interest.

## Publisher’s Note

All claims expressed in this article are solely those of the authors and do not necessarily represent those of their affiliated organizations, or those of the publisher, the editors and the reviewers. Any product that may be evaluated in this article, or claim that may be made by its manufacturer, is not guaranteed or endorsed by the publisher.

## Acknowledgments

We thank Casa Nossa Senhora da Paz for the financial support with the publication fee.

## Supplementary Material

The Supplementary Material for this article can be found online at: https://www.frontiersin.org/articles/10.3389/fmicb.2022.898815/full#supplementary-material
