# Supplementary material for: Influence of the Polysaccharide Capsule on the Bactericidal Activity of Indolicidin on Streptococcus pneumoniae
Source: Front Microbiol. 2022 May 13;13:898815. doi: 10.3389/fmicb.2022.898815 (PMC9136410; doi:10.3389/fmicb.2022.898815)
Supplement: Supplementary file 1 [file Data_Sheet_1.PDF]

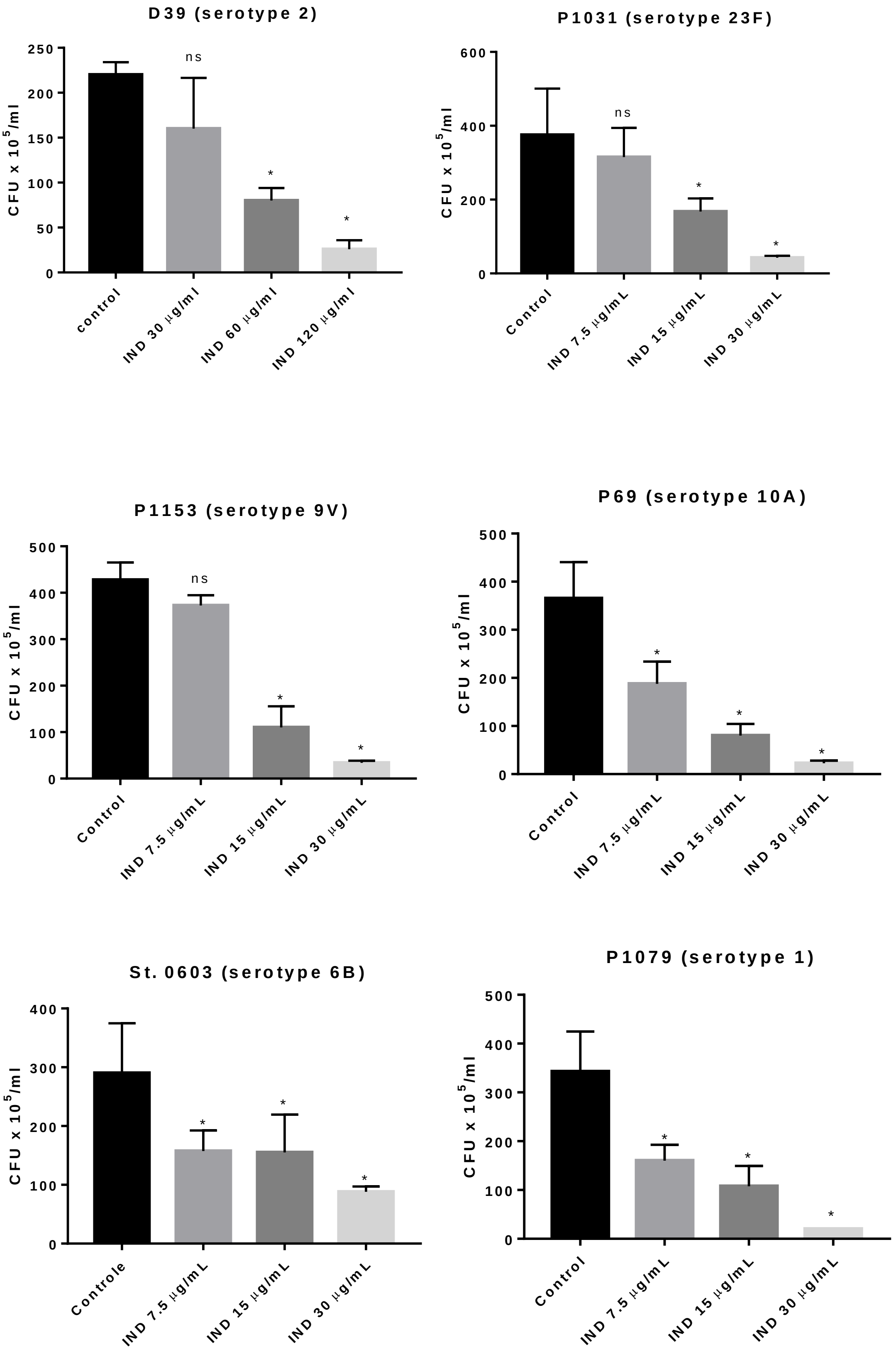
D39 (serotype 2)
CFU x 10^5/ml
250
200
150
100
50
0
ns
*
*
control
IND 30 µg/ml
IND 60 µg/ml
IND 120 µg/ml
P1031 (serotype 23F)
CFU x 10^5/ml
600
400
200
0
ns
*
*
Control
IND 7.5 µg/mL
IND 15 µg/mL
IND 30 µg/mL
P1153 (serotype 9V)
CFU x 10^5/ml
500
400
300
200
100
0
ns
*
*
Control
IND 7.5 µg/mL
IND 15 µg/mL
IND 30 µg/mL
P69 (serotype 10A)
CFU x 10^5/ml
500
400
300
200
100
0
*
*
*
Control
IND 7.5 µg/mL
IND 15 µg/mL
IND 30 µg/mL
St. 0603 (serotype 6B)
CFU x 10^5/ml
400
300
200
100
0
*
*
*
Controle
IND 7.5 µg/mL
IND 15 µg/mL
IND 30 µg/mL
P1079 (serotype 1)
CFU x 10^5/ml
500
400
300
200
100
0
*
*
*
Control
IND 7.5 µg/mL
IND 15 µg/mL
IND 30 µg/mL

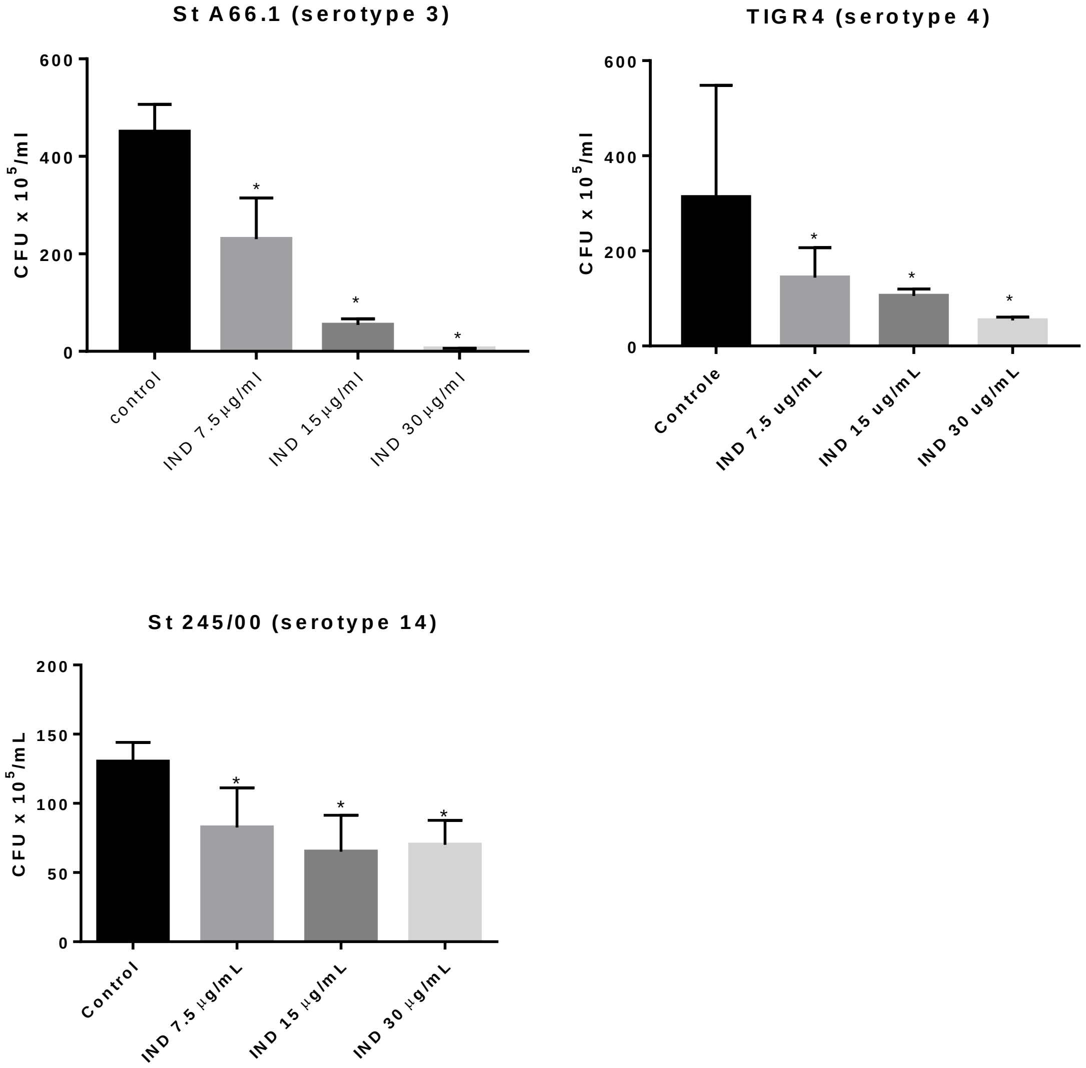

Supplementary figure 1. Susceptibility of different *Streptococcus pneumoniae* serotypes to indolicidin. Bacterial strains were treated with increasing concentrations of indolicidin and plated. The control group was incubated with PBS only. The number of bacteria surviving treatment is shown for each condition. Statistical analysis was performed using ANOVA with a Dunnet post-test. *$p<0.05$ in comparison with control.
